# Supplementary figures and images for: Transcriptomic profiling of susceptible and resistant flax seedlings after Fusarium oxysporum lini infection
Source: PLoS One. 2021 Jan 26;16(1):e0246052. doi: 10.1371/journal.pone.0246052 (PMC7837494; doi:10.1371/journal.pone.0246052)

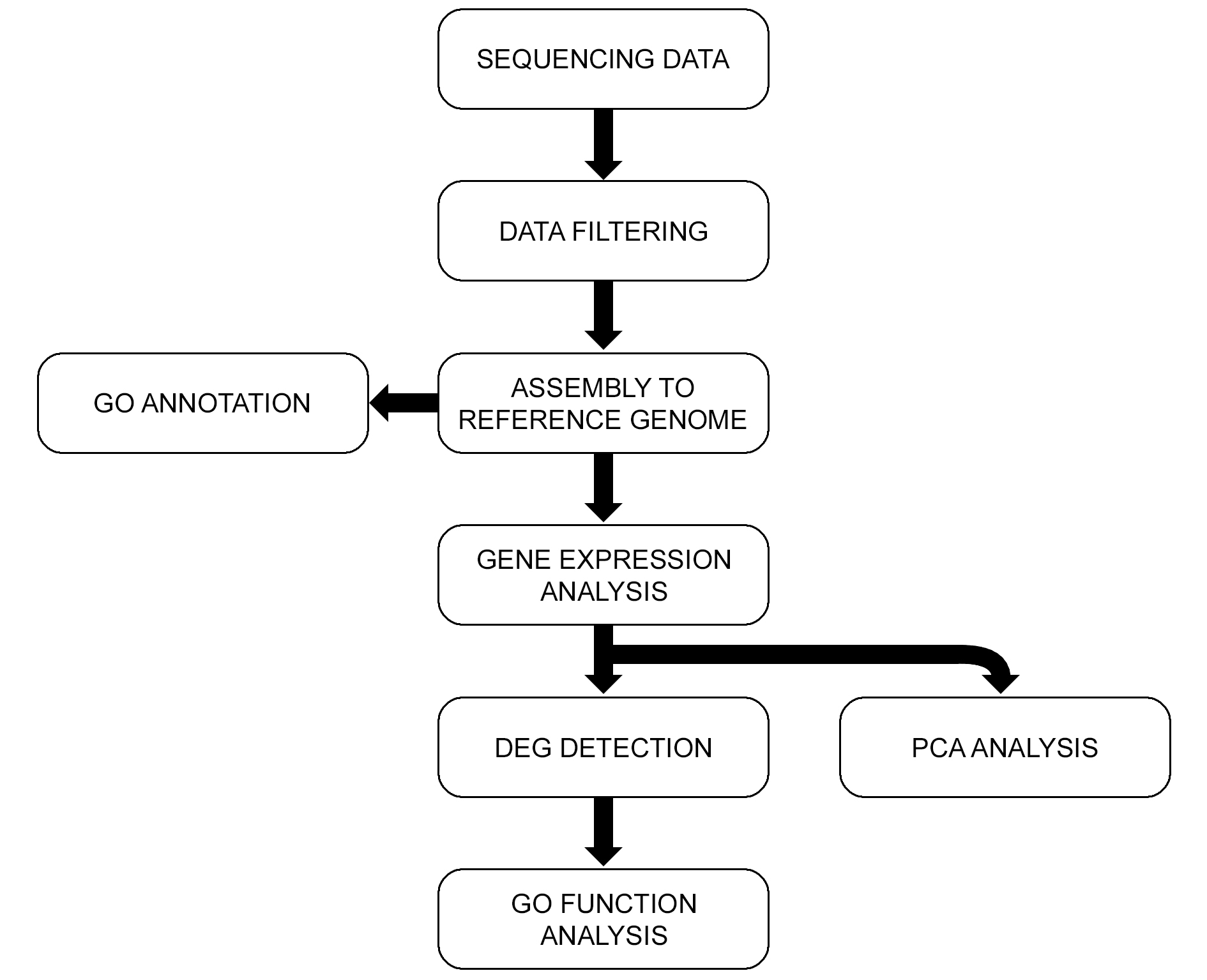

Supplement: S1 Fig — (TIF) [file pone.0246052.s001.tif]

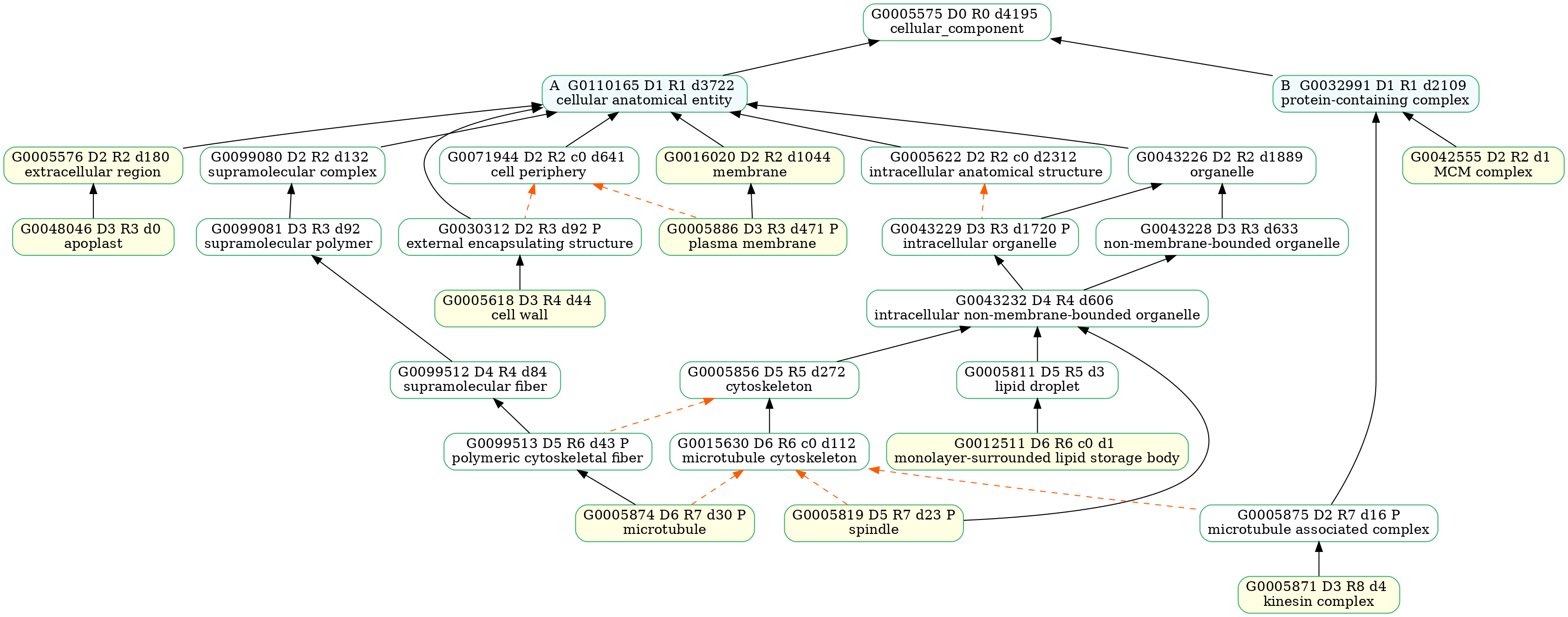

Supplement: S2 Fig — (ZIP) [file pone.0246052.s002.zip › NIKE_24h_CTRL_vs_Fol_.GO.CC.png]

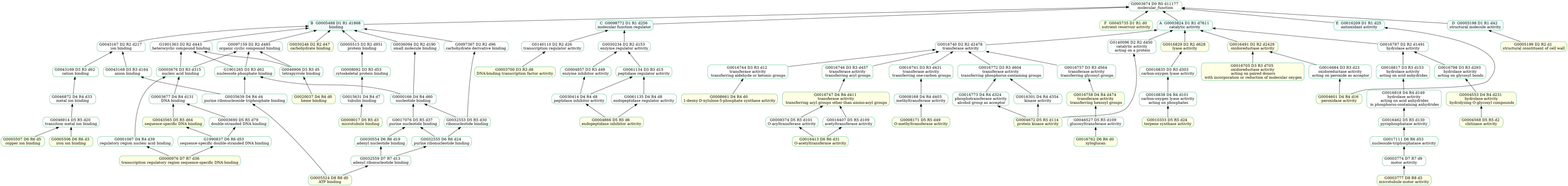

Supplement: S2 Fig — (ZIP) [file pone.0246052.s002.zip › NIKE_24h_CTRL_vs_Fol_.GO.MF.png]

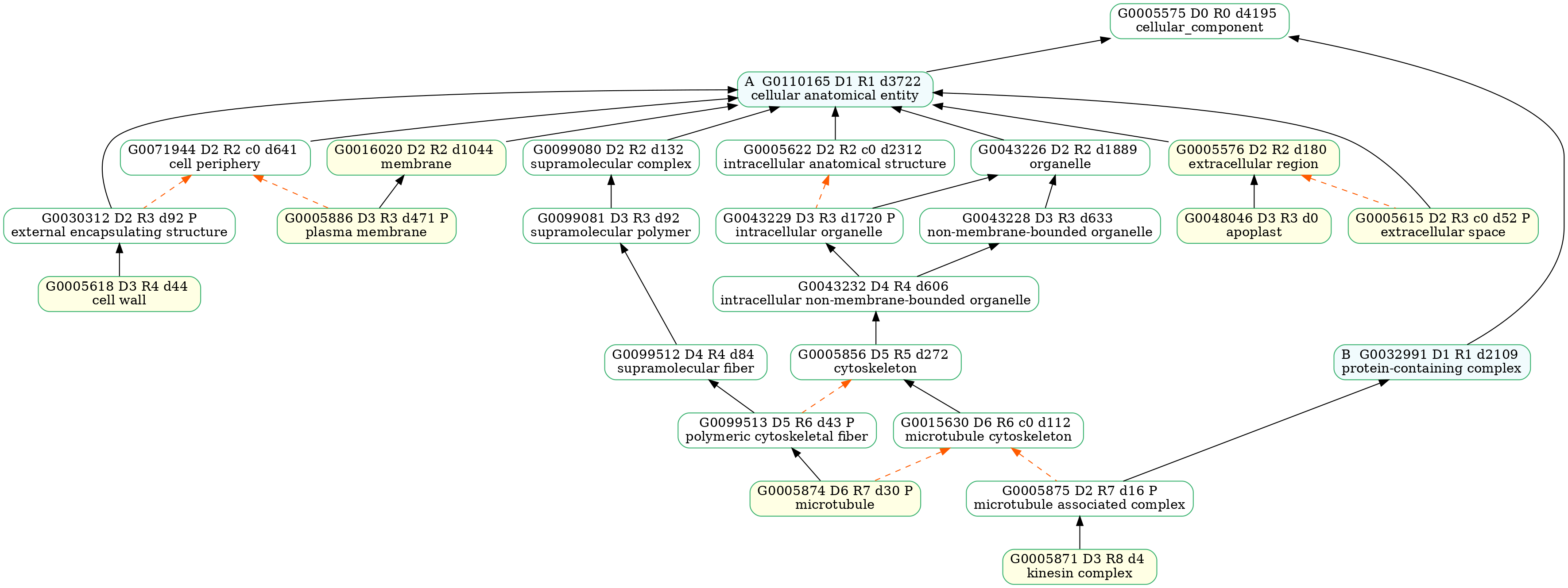

Supplement: S2 Fig — (ZIP) [file pone.0246052.s002.zip › NIKE_48h_CTRL_vs_Fol_.GO.CC.png]

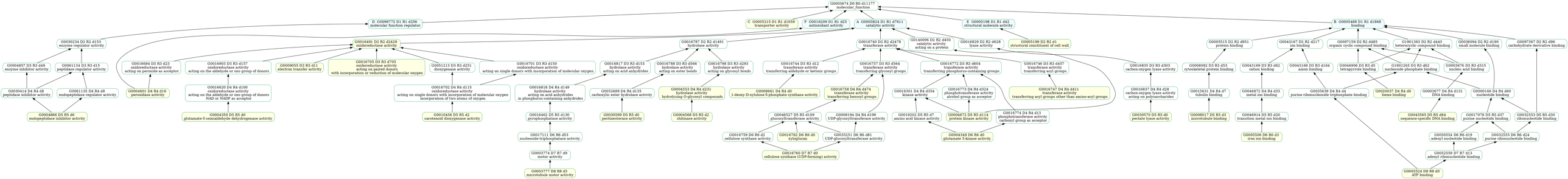

Supplement: S2 Fig — (ZIP) [file pone.0246052.s002.zip › NIKE_48h_CTRL_vs_Fol_.GO.MF.png]

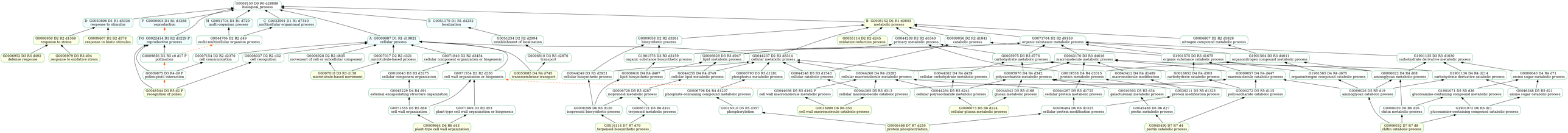

Supplement: S2 Fig — (ZIP) [file pone.0246052.s002.zip › REGINA_24h_CTRL_vs_Fol_.GO.BP.png]

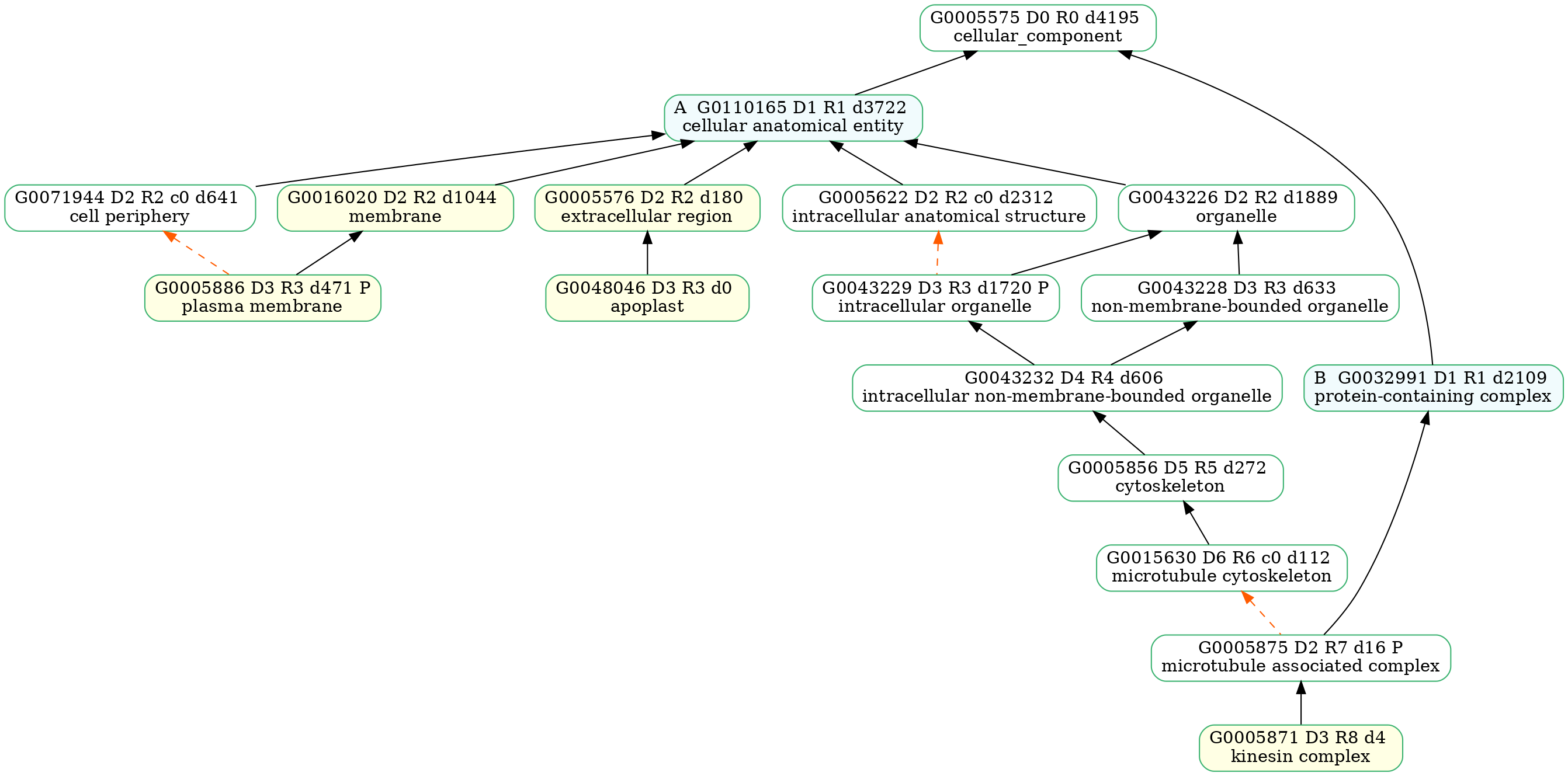

Supplement: S2 Fig — (ZIP) [file pone.0246052.s002.zip › REGINA_24h_CTRL_vs_Fol_.GO.CC.png]

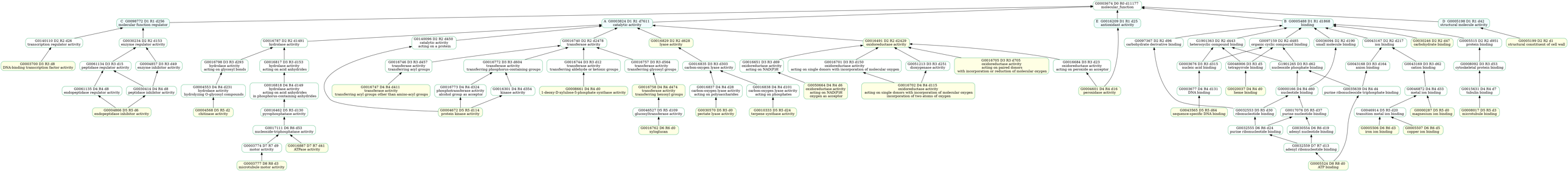

Supplement: S2 Fig — (ZIP) [file pone.0246052.s002.zip › REGINA_24h_CTRL_vs_Fol_.GO.MF.png]

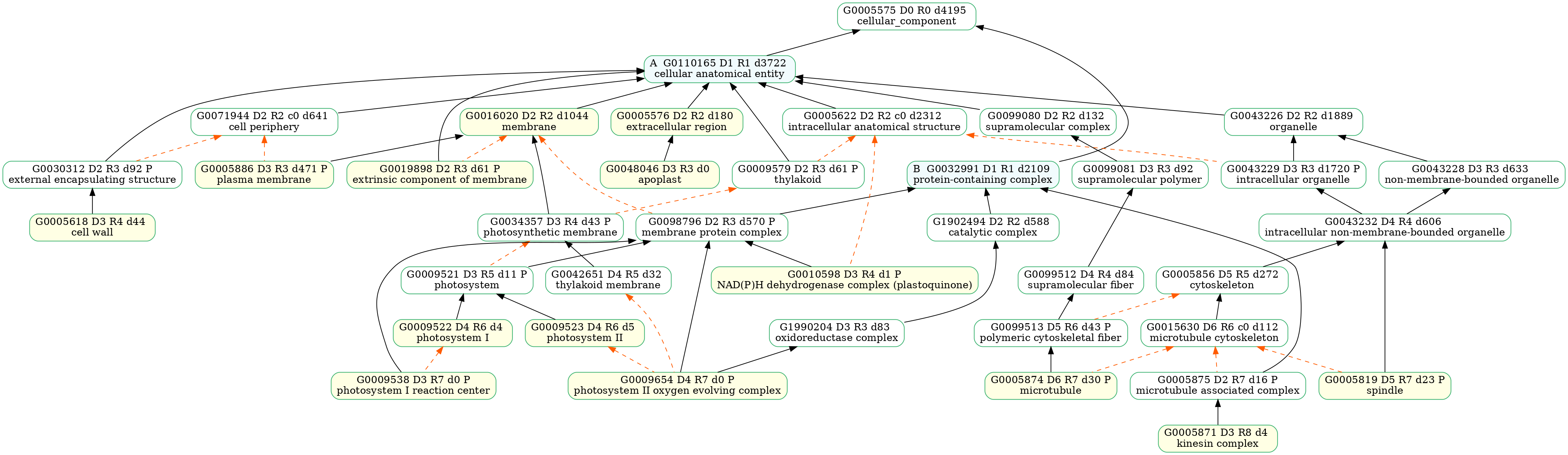

Supplement: S2 Fig — (ZIP) [file pone.0246052.s002.zip › REGINA_48h_CTRL_vs_Fol_.GO.CC.png]

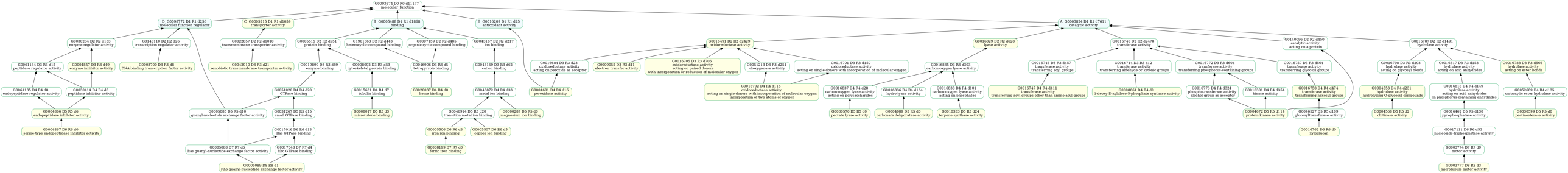

Supplement: S2 Fig — (ZIP) [file pone.0246052.s002.zip › REGINA_48h_CTRL_vs_Fol_.GO.MF.png]
