# Supplementary material for: Transcriptomic profiling of susceptible and resistant flax seedlings after Fusarium oxysporum lini infection
Source: PLoS One. 2021 Jan 26;16(1):e0246052. doi: 10.1371/journal.pone.0246052 (PMC7837494; doi:10.1371/journal.pone.0246052)
Supplement: S1 Table — (DOCX) [file pone.0246052.s003.docx]

S1 Table. Sample Test Result.

| No. | Sample Name | Sample Number | Tube No. | Conc. (ng/ μL) | Vol. (μL) | Total Mass (μg) | OD260/280 | OD260/230 | RIN | 28S/ 18S | Library Type | Test Result |
| --- | --- | --- | --- | --- | --- | --- | --- | --- | --- | --- | --- | --- |
| 1 | 1-1 | 8521805004183 | 1 | 980 | 12 | 11.76 | 2.1 | 2.17 | 9.4 | 2 | HiSeq Transcriptome | Qualified |
| 2 | 1-2 | 8521805004184 | 1 | 1300 | 12 | 15.6 | 2.1 | 2.18 | 9.3 | 2 | HiSeq Transcriptome | Qualified |
| 3 | 1-3 | 8521805004185 | 1 | 1440 | 12 | 17.28 | 2.07 | 2.12 | 9.2 | 1.9 | HiSeq Transcriptome | Qualified |
| 4 | 2-1 | 8521805004186 | 1 | 2300 | 12 | 27.6 | 2.16 | 2.1 | 8.2 | 1.7 | HiSeq Transcriptome | Qualified |
| 5 | 2-2 | 8521805004187 | 1 | 1920 | 12 | 23.04 | 2.12 | 2.09 | 7.6 | 1.7 | HiSeq Transcriptome | Qualified |
| 6 | 2-3 | 8521805004188 | 1 | 2090 | 12 | 25.08 | 2.14 | 2.08 | 7.6 | 1.6 | HiSeq Transcriptome | Qualified |
| 7 | 3-1 | 8521805004189 | 1 | 1190 | 17 | 20.23 | 2.11 | 1.86 | 9.3 | 1.9 | HiSeq Transcriptome | Qualified |
| 8 | 3-2 | 8521805004190 | 1 | 860 | 17 | 14.62 | 2.09 | 1.88 | 9.2 | 1.9 | HiSeq Transcriptome | Qualified |
| 9 | 3-3 | 8521805004191 | 1 | 870 | 17 | 14.79 | 2.11 | 1.89 | 9.2 | 1.9 | HiSeq Transcriptome | Qualified |
| 10 | 4-1 | 8521805004192 | 1 | 1000 | 17 | 17 | 2.12 | 1.74 | 7.6 | 2.1 | HiSeq Transcriptome | Qualified |
| 11 | 4-2 | 8521805004193 | 1 | 990 | 17 | 16.83 | 2.11 | 1.67 | 7.8 | 2.1 | HiSeq Transcriptome | Qualified |
| 12 | 4-3 | 8521805004194 | 1 | 930 | 17 | 15.81 | 2.1 | 1.69 | 8 | 2.1 | HiSeq Transcriptome | Qualified |
| 13 | 5-1 | 8521805004195 | 1 | 680 | 17 | 11.56 | 2.06 | 2.1 | 9.1 | 2.1 | HiSeq Transcriptome | Qualified |
| 14 | 5-2 | 8521805004196 | 1 | 590 | 17 | 10.03 | 2.06 | 2.05 | 9.2 | 2.1 | HiSeq Transcriptome | Qualified |
| 15 | 5-3 | 8521805004197 | 1 | 730 | 17 | 12.41 | 2.1 | 2.13 | 9.1 | 2.1 | HiSeq Transcriptome | Qualified |
| 16 | 6-1 | 8521805004198 | 1 | 650 | 17 | 11.05 | 2.08 | 1.65 | 9 | 2 | HiSeq Transcriptome | Qualified |
| 17 | 6-2 | 8521805004199 | 1 | 810 | 17 | 13.77 | 2.08 | 1.67 | 8.9 | 2.1 | HiSeq Transcriptome | Qualified |
| 18 | 6-3 | 8521805004200 | 1 | 610 | 17 | 10.37 | 2.06 | 1.57 | 8.9 | 2 | HiSeq Transcriptome | Qualified |
| 19 | 7-1 | 8521805004201 | 1 | 800 | 17 | 13.6 | 2.09 | 1.57 | 8.3 | 2 | HiSeq Transcriptome | Qualified |
| 20 | 7-2 | 8521805004202 | 1 | 680 | 17 | 11.56 | 2.1 | 1.55 | 8.7 | 2 | HiSeq Transcriptome | Qualified |
| 21 | 7-3 | 8521805004203 | 1 | 730 | 17 | 12.41 | 2.07 | 1.56 | 8.8 | 2.1 | HiSeq Transcriptome | Qualified |
| 22 | 8-1 | 8521805004204 | 1 | 1030 | 17 | 17.51 | 2.09 | 1.77 | 8.4 | 1.9 | HiSeq Transcriptome | Qualified |
| 23 | 8-2 | 8521805004205 | 1 | 910 | 17 | 15.47 | 2.08 | 1.67 | 8.3 | 1.8 | HiSeq Transcriptome | Qualified |
| 24 | 8-3 | 8521805004206 | 1 | 850 | 17 | 14.45 | 2.1 | 1.64 | 8.1 | 1.8 | HiSeq Transcriptome | Qualified |
